# Possible involvement of caveolin in attenuation of cardioprotective effect of ischemic preconditioning in diabetic rat heart

**DOI:** 10.1186/1471-2261-11-43

**Published:** 2011-07-12

**Authors:** Preeti Ajmani, Harlokesh N Yadav, Manjeet Singh, Pyare L Sharma

**Affiliations:** 1 Department of Pharmacology, Indo-Soviet College of Pharmacy, Moga 142-001, Punjab, India

## Abstract

**Background:**

Nitric oxide (NO) has been noted to produce ischemic preconditioning (IPC)-mediated cardioprotection. Caveolin is a negative regulator of NO, which inhibits endothelial nitric oxide synthase (eNOS) by making caveolin-eNOS complex. The expression of caveolin is increased during diabetes mellitus (DM). The present study was designed to investigate the involvement of caveolin in attenuation of the cardioprotective effect of IPC during DM in rat.

**Methods:**

Experimental DM was induced by single dose of streptozotocin (50 mg/Kg, *i.p*,) and animals were used for experiments four weeks later. Isolated heart was mounted on Langendorff's apparatus, and was subjected to 30 min of global ischemia and 120 min of reperfusion. IPC was given by four cycles of 5 min of ischemia and 5 min of reperfusion with Kreb's-Henseleit solution (K-H). Extent of injury was measured in terms of infarct size by triphenyltetrazolium chloride (TTC) staining, and release of lactate dehydrogenase (LDH) and creatin kinase-MB (CK-MB) in coronary effluent. The cardiac release of NO was noted by measuring the level of nitrite in coronary effluent.

**Results:**

IPC- induced cardioprotection and release of NO was significantly decreased in diabetic rat heart. Pre-treatment of diabetic rat with daidzein (DDZ) a caveolin inhibitor (0.2 mg/Kg/s.c), for one week, significantly increased the release of NO and restored the attenuated cardioprotective effect of IPC. Also perfusion of sodium nitrite (10 μM/L), a precursor of NO, significantly restored the lost effect of IPC, similar to daidzein in diabetic rat. Administration of 5-hydroxy deaconate (5-HD), a mito K_ATP _channel blocker, significantly abolished the observed IPC-induced cardioprotection in normal rat or daidzein and sodium nitrite perfused diabetic rat heart alone or in combination.

**Conclusions:**

Thus, it is suggested that attenuation of the cardioprotection in diabetic heart may be due to decrease the IPC mediated release of NO in the diabetic myocardium, which may be due to up -regulation of caveolin and subsequently decreased activity of eNOS.

## Background

Ischemic heart disease is a leading cause of morbidity and mortality worldwide [1]. Reperfusion of an ischemic myocardium is a requisite, for the restoration of the normal functioning of the myocardium [2]. However, abrupt reperfusion of an ischemic myocardium is not without hazard; it produces further damage of myocardium, described as ischemia-reperfusion (I/R) injury [3,4]. Moreover, it has been documented that "controlled reperfusion" avoids further injury, both in myocardium and in peripheral tissues [5-8]. Brief episodes of ischemia followed by reperfusion of myocardium, increase the resistance against sustained ischemia of longer duration; this phenomenon is termed as ischemic preconditioning (IPC) [9]. IPC produces cardioprotection by PI-3K/Akt [10,11], phosphorylation of eNOS and by generation of nitric oxide (NO) and by opening of mito K_ATP _channel [12,13]. However, the cardioprotective effect of IPC is attenuated in conditions such as heart failure [14,15] aging [16,17] hypertension ([18,19] obesity [20] hyperlipidemia [21-23]and diabetes mellitus [24-26]. Diabetes mellitus is a one of the major risk factor for ischemic heart disease.

Caveolin is the caveolar membrane protein, invaginated on the plasma membrane that serves as signalling platform for many of the G-protein coupled receptors (GPCR) [27-29]. IPC exerts cardioprotection by impairing the death signalling components p38MAPKα and JNK [30], by increase its association with caveolin. It has been well documented that caveolin is a negative regulator of eNOS, it interacts and inhibits the activity of eNOS by making caveolin-eNOS complex [31,32]. IPC increases the activity of eNOS by disrupting the complex of caveolin and eNOS in rat heart [32]. Moreover, it has been documented that NO produces cardioprotection by opening of K_ATP _channel during IPC, and caveolin facilitates the interaction of NO with K_ATP _channel by forming a suitable signaling platform [33]. Caveolin maintains eNOS in inactivated state and thereby limits NO production [34,35] and on agonist stimulation leads to activation of eNOS through increased disruption of caveolin/eNOS complex [31].

In diabetic rat heart, expression of caveolin increases [36-38] which enhances the binding of eNOS to caveolin and decreases the release of NO [31]. Therefore, the present study was undertaken to elucidate whether or not the diminished eNOS/NO signaling in diabetic myocardium is responsible for loss of cardioprotective effect of IPC.

## Methods

The experimental protocol used in the present study was approved by Institutional Animal Ethics Committee.

### Drugs and chemicals

Daidzein (0.2 mg/Kg/s.c) (Sigma Aldrich [P] Ltd., Bangalore, India) was dissolved in dimethyl sulfoxide (DMSO) and then injected to the animals for 7 days, 3 weeks after the administration of streptozotocin. Sodium nitrite (10 μM/L) (Rankem, Fine Chemicals Ltd., New Delhi, India) and 5-Hydroxy Decanoate (100 μM/L) (Sigma Aldrich [P] Ltd., Bangalore, India) were added in minimum quantity of distilled water and added to Kreb's Henseleit solution. All other reagents used in this study were of analytical grade and always freshly prepared before use.

### Induction of experimental diabetes

Total 12 groups have been used in present study each group consist of 6-10 Wistar rats (180-250) of either sex. Experimental diabetes was induced by single dose administration of streptozotocin (50 mg/kg, *i.p*) [39]. There was 10% of mortality within 1^st ^week and 20% mortality was noted up to harvesting of heart. Serum glucose was estimated spectrophotometrically at 505 nm by glucose oxidase/pyruvate oxidase (GOD-POD) method [40,41] using an enzymatic kit (Kamineni Life Sciences Pvt. Ltd. Hyderabad, India). Serum glucose level > 200 mg/dl were considered to be hyperglycaemic.

### Isolated rat heart preparation

Rats were administered heparin (500 IU/L, i.p) 20 min. prior to sacrificing the animal by cervical dislocation. Heart was rapidly excised and immediately mounted on Langendorff's apparatus [42]. Isolated heart was retrogradely perfused at constant pressure of 80 mmHg with Kreb's-Henseleit (KH) buffer (NaCl 118 mM; KCl 4.7 mM; CaCl2 2.5 mM; MgSO4.7H20 1.2 mM; KH2PO4 1.2 mM; C6H12O6 11 mM), pH 7.4, maintained at 37°C bubbled with 95% O2 and 5%CO2. Flow rate was maintained at 7-9 ml/min. using Hoffman's screw. The heart was enclosed in double wall jacket, the temperature of which was maintained by circulating water heated at 37°C. Ischemic preconditioning was produced by closing the inflow of K-H solution for 5 min followed by 5 min of reperfusion. Four such episodes were employed. Global ischemia was produced for 30 min. followed by 120 min. of reperfusion. Coronary effluent was collected before ischemia, immediately, 5 min. and 30 min. after reperfusion for estimation of Lactate Dehydrogenase (LDH) and Creatine Kinase (CK-MB) [43].

### Assessment of myocardial injury

The assessment of myocardial infarct size was done by using triphenyltetrazolium chloride (TTC) staining method, while LDH and CK-MB were estimated by using commercially available kits (LDH Siemens Medical Solution Diagnostics Ltd., Ajwa Road, Baroda, India, CK-MB Nicholas Piramal India Ltd., Mumbai). Values of LDH and CK-MB were expressed in international units per litre (IU/L).

### Assessment of myocardial infarct size

The heart was removed from the Langendorff's apparatus. Both the atria and root of aorta were excised and ventricles were kept overnight at -4°C temperature. Frozen ventricles were sliced into uniform sections of about 1-2 mm thickness. The slices were incubated in 1% w/v triphenyltetrazolium chloride stain (TTC stain) at 37°C in 0.2 M Tris-chloride buffer for 30 min. The normal myocardium was stained brick red while the infarcted portion remained unstained. Infarct size was measured by the volume method [44]

### Nitrite estimation

Nitrite is a stable nitrogen intermediate formed from the spontaneous degradation of NO. Unlike NO, nitrite can be measured easily and nitrite concentrations can be used to infer levels of NO production [45-47]. Nitrite release in coronary effluent was measured [48]. Greiss reagent 0.5 ml (1:1 solution of 1% sulphanilamide in 5% phosphoric acid and 0.1% N-(1-Naphthyl) ethylenediamine dihydrochloride in water) was added to 0.5 ml of coronary effluent. The optical density at 550 nm was measured using spectrophotometer (UV-1700 Spectrophotometer, Shimadzu, Japan). Nitrite concentration was calculated by comparison with spectrophotometer reading of standard solution of sodium nitrite prepared in K-H buffer [48]

### Experimental protocol

**Figure 1:**
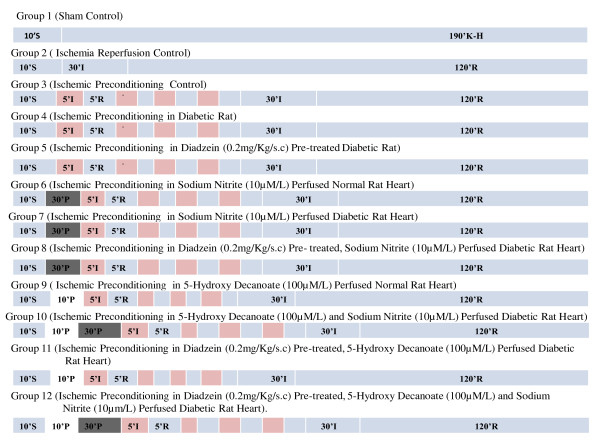
**Diagrammatic representation of experimental protocol**. S, P, I, R, denotes stabilization, perfusion, ischemia and reperfusion

A diagrammatic representation of experimental protocol is shown in Figure 1. In all groups, isolated rat heart was perfused with K-H solution and allowed for 10 min of stabilization. Group 1 (Sham Control; n = 6): Isolated rat heart was perfused continuously for 200 min without subjecting them to global ischemia and reperfusion. Group 2 (Ischemia-Reperfusion Control; n = 6): After 10 min of stabilization, isolated rat heart preparation was subjected to 30 min. global ischemia followed by 120 min. of reperfusion. Group 3 (Ischemic Preconditioning Control; n = 6): After 10 min of stabilization, heart was subjected to four cycles of ischemic preconditioning, each cycle comprised of 5 min. global ischemia followed by 5 min. reperfusion further followed by 30 min. global ischemia and 120 min. of reperfusion. Group 4 (Ischemic Preconditioning in Diabetic Rats; n = 6): Isolated heart preparation from diabetic rat subjected to four cycles of ischemic preconditioning as described earlier in group 3. Group 5 (Ischemic Preconditioning in Daidzein (0.2 mg/Kg/s.c/day) Pre-treated Diabetic Rat; n = 6): Isolated rat heart preparation from daidzein (0.2 mg/Kg/s.c/day) pre-treated diabetic rat was subjected to four cycles of ischemic preconditioning as described earlier in group 3. Group 6 (Ischemic preconditioning in Sodium Nitrite (10 μM/L) perfused Normal Rat Heart; n = 6): After 10 min of stabilization, heart was perfused with K-H buffer containing sodium nitrite (10 μM/L) for 30 min. and then subjected to four cycles of ischemic preconditioning as described earlier in group 3. Group 7 (Ischemic preconditioning in Sodium Nitrite (10 μM/L) perfused Diabetic Rat Heart; n = 6): Isolated heart preparation obtained from diabetic rat was perfused with sodium nitrite (10 μM/L) for 30 min. followed by IPC as described in group 3. Group 8 (Ischemic Preconditioning in Daidzein (0.2 mg/Kg/s.c/day) Pre-treated, Sodium Nitrite (10 μM/L) Perfused Diabetic Rat Heart; n = 6): After 10 min of stabilization, Isolated rat heart preparation from daidzein (0.2 mg/Kg/s.c/day) pre-treated diabetic rat was perfused with sodium nitrite (10 μM/L) for 30 min. followed by as described in group 3. Group 9 (Ischemic Preconditioning in 5-Hydroxy Decanoate (100 μM/L) Perfused Normal Rat Heart; n = 6): After 10 min of stabilization, isolated heart was perfused with K-H buffer containing 5-hydroxy decanoate (100 μM/L) for 10 min. and then subjected to IPC as described earlier in group 3. Group 10 (Ischemic Preconditioning in Daidzein (0.2 mg/Kg/s.c/day) Pre-treated, 5-Hydroxy Decanoate (100 μM/L) Perfused Diabetic Rat Heart; n = 6): After 10 min of stabilization, isolated rat heart preparation from daidzein (0.2 mg/Kg/s.c/day) pre-treated diabetic rat perfused with 5-hydroxy decanoate (100 μM/L) containing K-H buffer for 10 min. followed by as described in group 3. Group 11 (Ischemic Preconditioning in 5-Hydroxy Decanoate (100 μM/L) and Sodium Nitrite (10 μM/L) Perfused Diabetic Rat Heart; n = 6): After 10 min of stabilization, isolated heart preparation from diabetic rat was perfused with K-H buffer containing 5-hydroxy decanoate (100 μM/L) for 10 min. which is further followed by perfusion with sodium nitrite (10 μM/L) for 30 min. followed by as described earlier in group 3. Group 12 (Ischemic Preconditioning in Daidzein (0.2 mg/Kg/s.c/day) Pre-treated, 5-Hydroxy Decanoate (100 μM/L) and Sodium Nitrite (10 μM/L) Perfused Diabetic Rat Heart; n = 6): After 10 min of stabilization, isolated rat heart preparation from daidzein (0.2 mg/Kg/s.c/day) treated diabetic rat was followedby group 11.

### Statistical analysis

All values were expressed as mean ± standard deviation (S.D). Statistical analysis was performed using Sigmastat Software. Glucose value was compared by Student's paired t-test. The values of infarct size, LDH, CK-MB and nitrite level were statistically analysed using one-way analysis of variance (ANOVA) followed by Tukey's multiple comparison test as a post hoc test. Value of P < 0.05 was considered to be statistically significant.

## Results

### Effect of streptozotocin on serum glucose

**Figure 2:**
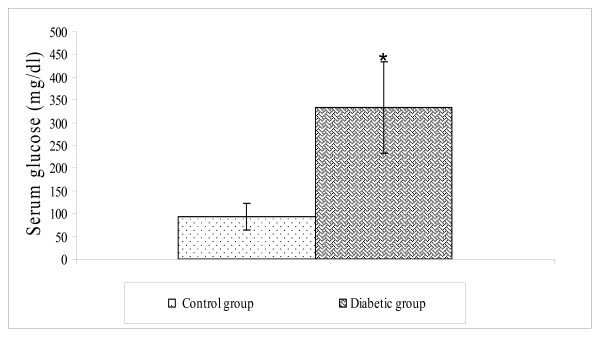
**Effect of streptozotocin administration on serum glucose in the rat**. Values are expressed as mean ± S.D. _* _= p < 0.05 vs. Control animals.

The administration of single dose of streptozotocin (50 mg/Kg, *i.p*) significantly increased blood glucose as compared to basal value (Figure 2).

### Effect of ischemic preconditioning and pharmacological interventions on myocardial infarct size

**Figure 3:**
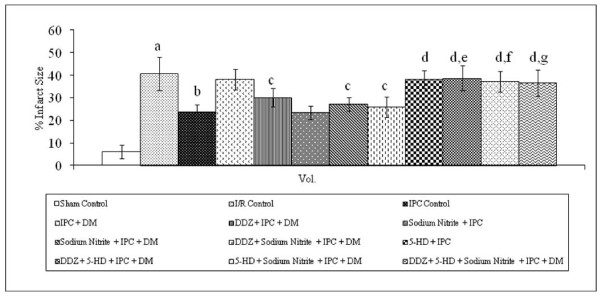
**Effect of I-R on myocardial infarct size, effect of ischemic preconditioning (IPC) on myocardial infarct size in normal and diabetic rat heart, effect of DDZ pre-treatment, sodium nitrite perfusion on myocardial infarct size in diabetic rat heart and effect of 5-HD alone or in combination with DDZ and sodium nitrite on myocardial infarct size in diabetic rat heart**. I/R, IPC, DM, DDZ, 5-HD denotes ischemia reperfusion, ischemic preconditioning, diabetes mellitus, daidzein and 5-hydroxy decanoate respectively. Values are expressed as mean ± S.D, a = p < 0.05 vs. sham control; b = p < 0.05 vs. I/R Control; c = p < 0.05 vs .IPC in diabetic rat heart; d = p < 0.05 vs. IPC in normal rat heart; e = P < 0.05 vs. IPC in DDZ pretreated diabetic rat heart; f = p < 0.05 vs. IPC in sodium nitrite perfused diabetic rat heart; g = p < 0.05 vs. IPC in DDZ pretreated, sodium nitrite perfused diabetic rat heart.

Global ischemia for 30 min followed by 120 min of reperfusion significantly increased the myocardial infarct size, as compared to sham control. Four episodes of IPC significantly decreased I/R induced increase in myocardial infarct size in normal rat heart. However, ischemic preconditioning failed to decrease the myocardial infarct size in diabetic rat heart. Moreover, IPC induced decrease of infarct size was significantly restored in DDZ pre-treated and in sodium nitrite perfused diabetic rat heart. However, perfusion with 5-HD significantly attenuated the decrease of myocardial infarct size in normal, DDZ pre-treated and sodium nitrite perfused diabetic rat heart alone or in combination (Figure 3).

### Effect of ischaemic preconditioning and pharmacological interventions on the release of Lactate dehydrogenase (LDH)

**Figure 4:**
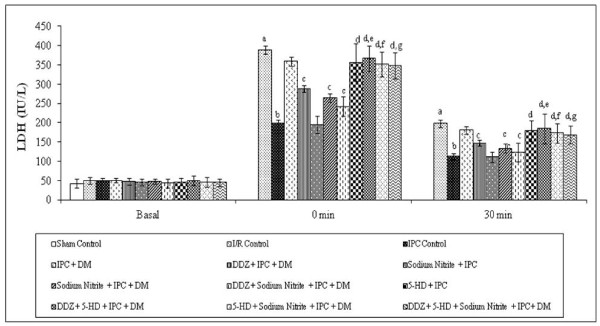
**Effect of I-R on the release of LDH, effect of ischemic preconditioning (IPC) on the release of LDH in normal and diabetic rat heart, effect of DDZ pre-treatment, sodium nitrite perfusion on the release of LDH in diabetic rat heart and effect of 5-HD alone or in combination with DDZ and sodium nitrite on the release of LDH in diabetic rat heart**. I/R, IPC, DM, DDZ, 5-HD denotes ischemia reperfusion, ischemic preconditioning, diabetes mellitus, daidzein and 5-hydroxy decanoate respectively. Values are expressed as mean ± S.D, a = p < 0.05 vs. sham control; b = p < 0.05 vs. I/R Control; c = p < 0.05 vs. IPC in diabetic rat heart; d = p < 0.05 vs. IPC in normal rat heart; e = P < 0.05 vs. IPC in DDZ pretreated diabetic rat heart; f = p < 0.05 vs. IPC in sodium nitrite perfused diabetic rat heart; g = p < 0.05 vs. IPC in DDZ pretreated, sodium nitrite perfused diabetic rat heart.

Global ischemia for 30 min followed by 120 min of reperfusion markedly increased the release of LDH as compared to sham control. Four episodes of IPC significantly reduced the I/R induced increase in the release of LDH in normal rat heart but not in the diabetic rat heart. Moreover, IPC induced decrease in the release of LDH was significantly restored in DDZ pre-treated, and in sodium nitrite perfused diabetic rat heart. However, perfusion with 5-HD significantly attenuated the decrease in the release of LDH in normal, DDZ pre-treated and sodium nitrite perfused diabetic rat heart alone or in combination (Figure 4).

### Effect of ischemic preconditioning and pharmacological interventions on the release of CK-MB

**Figure 5:**
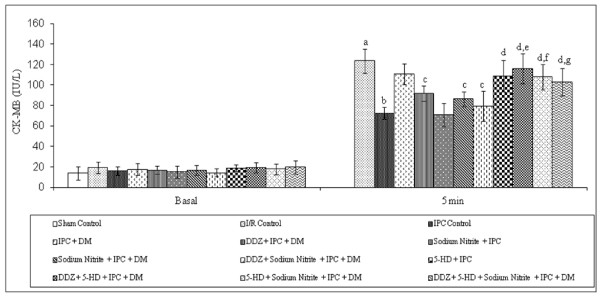
**Effect of I-R on the release of CK-MB, effect of ischemic preconditioning (IPC) on the release of CK-MB in normal and diabetic rat heart, effect of DDZ pre-treatment, sodium nitrite perfusion on the release of CK-MB in diabetic rat heart and effect of 5-HD alone or in combination with DDZ and sodium nitrite on the release of CK-MB in diabetic rat heart**. I/R, IPC, DM, DDZ, 5-HD denotes ischemia reperfusion, ischemic preconditioning, diabetes mellitus, daidzein and 5-hydroxy decanoate respectively. Values are expressed as mean ± S.D, a = p < 0.05 vs. sham control; b = p < 0.05 vs. I/R Control; c = p < 0.05 vs .IPC in diabetic rat heart; d = p < 0.05 vs. IPC in normal rat heart; e = P < 0.05 vs. IPC in DDZ pretreated diabetic rat heart; f = p < 0.05 vs. IPC in sodium nitrite perfused diabetic rat heart; g = p < 0.05 vs. IPC in DDZ pretreated, sodium nitrite perfused diabetic rat heart.

Global ischemia for 30 min followed by 120 min of reperfusion markedly increased the release of CK-MB, as compared to sham control. Four episodes of IPC significantly reduced the I/R induced increase in the release of CK-MB in normal rat heart but not in the diabetic rat heart. Moreover, IPC induced decrease release of CK-MB was significantly restored in DDZ pre-treated and sodium nitrite perfused diabetic rat heart. However, perfusion with 5-HD significantly attenuated the decrease in the release of CK-MB in normal, DDZ pre-treated and sodium nitrite perfused diabetic rat heart alone or in combination (Figure 5).

### Effect of ischemic preconditioning and treatment with daidzein on the release of nitrite

**Figure 6:**
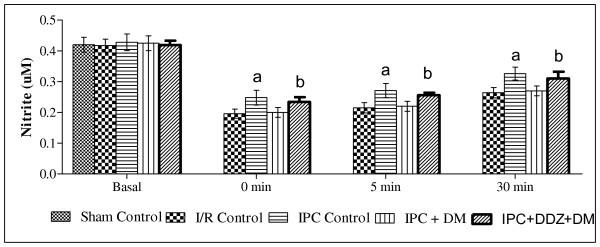
**Effect of ischemic preconditioning (IPC) and treatment of DDZ, on the release of nitric oxice in diabetic rat heart**. I/R, IPC, DM, DDZ, denotes ischemia reperfusion, ischemic preconditioning, diabetes mellitus and daidzein respectively. Values are expressed as mean ± S.D, a = p < 0.05 vs. I/R Control; b = p < 0.05 vs .IPC in diabetic rat heart.

Four episodes of IPC significantly increased the release of nitrite into coronary effluent of normal animals, as compared to I/R group but not in isolated heart obtained from diabetic rat. Treatment with daidzein, a caveolin inhibitor (0.2 mg/Kg/s.c, one week), significantly increased, the release of nitrite in diabetic rat heart subjected to IPC (Figure 6).

## Discussion

Four episodes of 5 min ischemia followed by reperfusion for 5 min, effectively preconditioned the normal rat heart as indicated by a significant decrease in infarct size and ischemia-reperfusion induced release of LDH and CK-MB. This cardioprotective effect of ischemic preconditioning is in agreement with earlier studies [49-51]. However the cardioprotective effect of ischemic preconditioning was significantly attenuated in diabetic rat heart. Our result is supported by earlier published studies [52-54,22,23]. It has been reported that perfusion of sodium nitrite produces cardioprotection in isolated heart from normal rat, subjected to global ischemia [55,56]. In our study, perfusion of sodium nitrite (a precursor of NO) [56] followed by IPC, significantly restored the attenuated effect of IPC in the diabetic myocardium (decreases in infarct size and in the release of LDH and CK-MB in coronary effluents). It is probable that the attenuated cardioprotective effect of IPC in diabetic rat may be due to decreased availability of NO. Thus, NO appears to be responsible for cardioprotective effect of ischemic preconditioning [13]. However, in our study, treatment with sodium nitrite did not enhance the cardioprotective effect of IPC in normal rat. This indicates that once IPC mediated increased generation of NO achieved the threshold for cardioprotection and the addition of sodium nitrite (NO donor) [56] was unable to further increase the myocardial protection by IPC, per se.

Opening of mitochondrial ATP sensitive potassium channels (mito K_ATP _channels) protects the myocardium from ischemia-reperfusion induced injury [57]. Various mediators i.e. adenosine, bradykinin, angiotensin, prostaglandins and NO which are released by the stimuli of IPC produce cardioprotection through opening of mito K_ATP _channel [58,59]. Further, administration of 5-HD, a mitoK_ATP _channel blocker, attenuated the observed cardioprotective effect of IPC in normal rat heart and in the sodium nitrite perfused diabetic rat heart. It is suggested that the observed cardioprotective effect of IPC in normal rat and sodium nitrite perfused diabetic ratmay be due to opening of mito K_ATP _channel. Our results are in accordance with reports from other laboratories [60,61].

Caveolins are proteins that form the structure of caveolar membrane, act as a signaling platform (signalosomes) for molecules such as nitric oxide synthase (NOS) and Src-like kinases and many of the G-proteins coupled receptors (GPCR) [27-29]. Ischemic preconditioning can modulate the microenvironment of caveolin and promote the signalling involved in protection of myocardium against ischemia-reperfusion induced injury [62]. It has been reported that expression of caveolin is upregulated in diabetic myocardium [37,38]. Caveolin is known to be a negative regulator of NO, it maintains eNOS in inactivated state by making caveolin-eNOS complex [36] and on agonist stimulation leads to activation of eNOS and increased release of NO; by disrupting caveolin/eNOS complex [31]. Increased expression of caveolin may lead to the increased interaction with eNOS, decreasing it's phosphorylation and a consequent decrease in the generation of NO [63]. In our study, IPC-induced release of nitrite in diabetic rat was significantly decreased as compared to normal rat. Our finding is supported by other reports that the decreased release of NO in the diabetic rat heart, is due to decreased activity of eNOS by an upregulation of caveolin in the myocardium [36,38].

Treatment with daidzein, has been noted to inhibit the expression of a caveolin in the diabetic rat [64,65]. In the present study daidzein treatment for seven days, three weeks after the administration of STZ, followed by IPC; significantly restored the cardioprotective effect in diabetic rat heart and increased the release of NO, without affecting the serum glucose. In normal heart, IPC leads to increased expression of caveolae [66]. Each brief episode of coronary occlusion produces small bursts of reactive oxygen species (ROS), and leads to increased formation and release of NO, by cleaving the caveolin-eNOS complex. Furthermore, antioxidants have been demonstrated to abolish IPC-induced cardiac protection in normal heart (62,67,68). Why IPC-induced cardiac protection is lost in diabetic heart is not known?. However, the restoration of IPC-induced cardiac protection by daidzein pre-treatment indicates that some defect in caveolin-eNOS complex may be involved in this process, as indicated in our study by a decrease in release of nitrite in the coronary effluent in the diabetic heart and its significant attenuation by daidzein pre-treatment. Roth and Patel (69) demonstrated that interaction of signalling molecules with caveolae is necessary for cardiac protection. The results obtained in our study support this viewpoint.

In the present study, we have used daidzein as an inhibitor of expression of caveolin in male as well as female rats. A limitation of daidzein, is that being a phytoesterogen it may modulate the estrogens status in female animals. However, in an earlier study, no significant difference in the effect of daidzein was detected between male and female rats (data not shown). Also, the restoration of the cardioprotective effect of IPC in diabetic rat heart by combination of daidzein and sodium nitrite was not greater than that observed when these drugs were used alone, suggesting thereby that these two drugs act by the same mechanism i.e., NO pathway.

## Conclusions

On the basis of above discussion, it may be concluded that attenuation of cardioprotective effect of ischemic preconditioning in diabetic rat heart is due to some defect in caveolin-eNOS complex in diabetic heart, which leads to, a decrease in the availability of NO and the consequent decreased activation of mito K_ATP _channels. Also, the IPC-induced changes in eNOS and NO in daidzein pre-treated diabetic heart, closely mimic those produced by IPC in the non-diseased heart.

## Limitation of the present study

Ideally, the proposed caveolin-eNOS interaction should have been assessed by coimmunoprecipitation study or by caveolin isolation.

## Competing interests

The authors declare that they have no competing interests.

## Authors' contributions

All authors, except MS approved the final manuscript. PA did most of the experimental work and data acquisition under the supervision of HNY, MS and PLS. HNY and PLS did the data analysis, interpretation and writing of the manuscript.

## Pre-publication history

The pre-publication history for this paper can be accessed here:

http://www.biomedcentral.com/1471-2261/11/43/prepub

## References

[B1] Murray CJ Lopez AD Alternative projections of mortality and disability by cause 1990-2020: Global burden of disease study Lancet 1997 349 9064 1498 1504 10.1016/S0140-6736(96)07492-2 9167458

[B2] Vandormael M Grines CL George BS Sanz ML Wall T O'Brien M Schwaiger M Aguirre FV The thrombolysis and angioplasty in myocardial infarction. A randomized trial of late reperfusion therapy for acute myocardial infarction Circulation 1992 85 6 2090 2099 1591828 10.1161/01.cir.85.6.2090

[B3] Baxter GF Ebrahim Z Role of bradykinin in preconditioning and protection of the ischaemic myocardium Br J Pharmacol 2002 135 4 843 854 10.1038/sj.bjp.0704548 11861312 PMC1573212

[B4] Piper HM Abdullah Y Schafer XXX The first minutes of reperfusion: a window of opportunity for cardioprotection Cardiovasc Res 2004 61 3 365 371 10.1016/j.cardiores.2003.12.012 14962469

[B5] Lindell SL Klahn SL Piazza TM Mangino MJ Torrealba JR Southard JH Carey HV Natural resistance to liver cold ischemia-reperfusion injury associated with the hibernation phenotype Am J Physiol Gastrointest Liver Physiol 2005 288 3 G473 G480 15701622 10.1152/ajpgi.00223.2004

[B6] Tsang A Hausenloy DJ Mocanu MM Yellon DM Postconditioning: a form of "Modified Reperfusion" protects the myocardium by activating the Phosphatidylinositol 3-Kinase-Akt Pathway Circ Res 2004 95 230 232 10.1161/01.RES.0000138303.76488.fe 15242972

[B7] Tsang A Hausenloy DJ Mocanu MM Carr RD Yellon DM Preconditioning the diabetic heart: the importance of Akt phosphorylation Diabetes 2005 54 8 2360 2364 10.2337/diabetes.54.8.2360 16046302

[B8] Mejía-Vilet JM Ramírez V Cruz C Uribe N Gamba G Bobadilla NA Renal ischemia-reperfusion injury is prevented by the mineralocorticoid receptor blocker spironolactone Am J Physiol Renal Physiol 2007 293 78 86 10.1152/ajprenal.00077.2007 17376767

[B9] Murry CE Jennings RB Reimer KA Preconditioning with ischemia: a delay of lethal cell injury in ischemic myocardium Circulation 1986 74 5 1124 1136 10.1161/01.CIR.74.5.1124 3769170

[B10] Stokoe D Stephens LR Copeland T Gaffney PR Reese CB Painter GF Holmes AB McCormick F Hawkins PT Dual Role of Phosphatidylinositol-3,4,5 trisphosphate in the activation of Protein Kinase B Science 1997 277 5325 567 570 10.1126/science.277.5325.567 9228007

[B11] Garg K Yadav HN Singh M Sharma PL Mechanism of Cardioprotective Effect of Erythropoietin-induced Preconditioning in Rat Heart Indian J Pharmacol 2010 42 4 219 223 10.4103/0253-7613.68421 20927246 PMC2941611

[B12] Ferdinandy P Schulz R Baxter GF Interaction of Cardiovascular Risk Factors with Myocardial Ischemia/Reperfusion Injury, Preconditioning and Postconditioning Pharmacol Rev 2007 59 4 418 458 10.1124/pr.107.06002 18048761

[B13] Prendes MGM Gonzalez M Savino EA Varela A Role of endogenous nitric oxide in classic preconditioning in rat hearts Regulatory Peptides 2007 139 1-3 141 145 10.1016/j.regpep.2006.10.015 17188373

[B14] Ferdinandy P Szilvassy Z Baxter GF Adaptation to myocardial stress in disease states: is preconditioning a healthy heart phenomenon? Trends Pharmacol Sci 1998 19 223 229 10.1016/S0165-6147(98)01212-7 9666713

[B15] Ferdinandy P Myocardial ischaemia/reperfusion injury and preconditioning: Effects of hypercholesterolaemia/hyperlipidaemia Br J Pharmacol 2003 138 2 283 285 10.1038/sj.bjp.0705097 12540517 PMC1573675

[B16] Abete P Ferrara N Cioppa A Ferrara P Bianco S Calabrese C Cacciatore F Longobardi G Rengo F Preconditioning does not prevent postischemic dysfunction in aging heart J Am Coll Cardiol 1996 27 7 1777 1786 10.1016/0735-1097(96)00070-8 8636568

[B17] Liu J Kam KWL Zhou J-J Yan W-Y Chen M Wu S Wong TM Effects of Heat Shock Protein 70 Activation by Metabolic Inhibition Preconditioning or Ðº- Opioid Receptor Stimulation on Ca^2+ ^Homeostasis in Rat Ventricular Myocytes Subjected to Ischemic Insults J Pharmacol Exp Ther 2004 310 606 613 10.1124/jpet.104.067926 15051801

[B18] Snoeckx LH Van Der Vuesse GJ Coumans WA Willemsen PH Reneman RS Differences in ischaemia tolerance between hypertrophied hearts of adult and aged spontaneously hypertensive rats Cardiovasc Res 1996 27 5 874 881 10.1093/cvr/27.5.874 8348587

[B19] Snoeckx LH Van Der Vusse GJ Coumans WA Willemsen PHM Van Der Nagel T Reneman RS Myocardial function in normal and spontaneously hypertensive rats during reperfusion after a period of global ischemia Cardiovasc Res 1986 20 1 67 75 10.1093/cvr/20.1.67 2939955

[B20] Sasaki H Ogawa K Shimizu M Mori C Takatsuka H Okazaki F Kawai M Taniguchi I Mochizuki S The insulin sensitizer pioglitazone improves the deterioration of ischemic preconditioning in Type 2 diabetes mellitus rats Int Heart J 2007 48 5 623 635 10.1536/ihj.48.623 17998772

[B21] Giricz Z Lalu MM Csonka C Bencsik P Schulz R Ferdinandy P Hyperlipidemia attenuates the infarct size-limiting effect of ischemic preconditioning: Role of matrix metalloproteinase-2 inhibition J Pharmacol Exp Ther 2006 316 1 154 161 16166272 10.1124/jpet.105.091140

[B22] Ungi I Ungi T Ruzsa Z Nagy E Zimmermann Z Csont T Ferdinandy P Hypercholesterolemia attenuates the anti-ischemic effect of preconditioning during coronary angioplasty Chest 2005 128 3 1623 1628 10.1378/chest.128.3.1623 16162767

[B23] Yadav H N Singh M Sharma PL Involvement of GSK-3β in Attenuation of The Cardioprotective Effect of Ischemic Preconditioning in Diabetic Rat Heart Mol Cell Biochem 2010 343 1-2 75 81 10.1007/s11010-010-0500-z 20512612

[B24] del Valle HF Lascano EC Negroni JA Ischemic preconditioning protection against stunning in conscious diabetic sheep: role of glucose, insulin, sarcolemmal and mitochondrial K_ATP _channels Cardiovasc Res 2002 55 3 642 659 10.1016/S0008-6363(02)00468-6 12160962

[B25] del Valle HF Lascano EC Negroni JA Crottogini AJ Absence of ischemic preconditioning protection in diabetic sheep hearts: role of sarcolemmal KATP Channel Dysfunction Mol Cell Biochem 2003 249 1-2 21 30 12956394

[B26] Yadav HN Singh M Sharma PL Modulation of the cardioprotective effect of ischemic preconditioning in hyperlipidaemic rat heart Eur J Pharmacol 2010 643 1 78 83 10.1016/j.ejphar.2010.06.015 20598682

[B27] Lisanti MP Scherer P Tang Z-L Sargiacomo M Caveolae, caveolin and caveolin-rich membrane domains: A signalling hypothesis Trends Cell Biol 1994 4 7 231 235 10.1016/0962-8924(94)90114-7 14731661

[B28] Couet J Sargiacomo M Lisanti MP Identification of peptide and protein ligands for the caveolin-scaffolding domain. Implications for the interaction of caveolin with caveolae-associated proteins J Biol Chem 1997 272 10 30429 30438 9045678 10.1074/jbc.272.10.6525

[B29] Song KS Sargiacomo M Galbiati F Parenti M Lisanti MP Targeting of subunit and c-Src tyrosine kinase to caveolae membranes: clarifying the role of N-myristoylation Cell Mol Biol 1997 43 3 293 303 9193783

[B30] Das M Cui J Das DK Generation of survival signal by differential interaction of p^38^MAPKα and p^38^MAPKβ with caveolin-1 and caveolin-3 in the adapted heart J Mol Cell Cardiol 2007 42 1 206 213 10.1016/j.yjmcc.2006.08.118 17069850 PMC2782735

[B31] Feron O Balligand JL Caveolin and the regulation of endothelial nitric oxide synthase in the heart Cardiovasc Res 2006 69 4 788 797 10.1016/j.cardiores.2005.12.014 16483868

[B32] Koneru S Penumathsa SV Thirunavukkarasu M Samuel SM Zhan L Han Z Maulik G Das DK Maulik N Redox regulation of ischemic preconditioning is mediated by the differential activation of caveolins and their association with eNOS and glut-4 Am J Physiol Heart Circ Physiol 2007 292 5 2060 72 10.1152/ajpheart.01169.2006 17277024

[B33] Garlid KD Costa ADT Quinlan CL Pierre SV Santos PD Cardioprotective signalling to mitochondria J Mol Cell Cardiol 2009 46 6 858 866 10.1016/j.yjmcc.2008.11.019 19118560 PMC2683183

[B34] Quinlan CL Costa ADT Costa CL Pierre SV Dos Santos P Garlid KD Conditioning the heart induces formation of signalosomes that interact with mitochondria to open MitoK_ATP_ Am J Physiol 2008 295 3 H953 61 10.1152/ajpheart.00520.2008 PMC2544503 18621853

[B35] Garcı'a-Carden˜ G Martasek P Masters BSS Skidd PM Coueti J Lii S Lisanti MP Sessa WC Dissecting the Interaction between Nitric Oxide Synthase (NOS) and Caveolin. Functional significance of the NOS caveolin binding domain in vivo J Biol Chem 1997 272 41 25437 25440 10.1074/jbc.272.41.25437 9325253

[B36] Maniatis NA Brovkovych V Allen SE John TA Shajahan AN Tiruppathi C Vogel SM Skidgel RA Malik AB Minshall RD Novel mechanism of endothelial nitric oxide synthase activation mediated by caveolae internalization in endothelial cells Circ Res 2006 99 8 870 877 10.1161/01.RES.0000245187.08026.47 16973909

[B37] Bucci M Roviezzo F Brancaleone V Lin MI Lorenzo AD Cicala C Pinto A Sessa WC Farneti S Fiorucci S Cirino G Diabetic mouse angiopathy is linked to progressive sympathetic receptor deletion coupled to an enhanced caveolin-1 expression Arterioscler Thromb Vasc Biol 2004 24 4 721 6 10.1161/01.ATV.0000122362.44628.09 14962949

[B38] Penumathsa SV Thirunavukkarasu M Zhan L Maulik G Menon VP Bagchi D Maulik N Resveratrol enhances GLUT-4 translocation to the caveolar lipid raft fractions through AMPK/AKT/eNOS signaling pathway in diabetic myocardium J Cell Mol Med 2008 12 6 2350 2361 10.1111/j.1582-4934.2008.00251.x 18266981 PMC4514113

[B39] Ozansoy G Akin FB Effects of gemfibrozil treatment on vascular reactivity of streptozotocin-diabetic rat aorta J Pharma Pharmacol 2004 56 2 241 246 10.1211/0022357022737 15005883

[B40] Trinder P Determination of glucose in blood using glucose oxidase with an alternative oxygen acceptor Ann Clin Biochem 1969 6 24 25

[B41] Lott J Turner K Evaluation of trinder's glucose oxidase method for measuring glucose in serum and urine Clin Chem 1975 21 12 1754 1760 1237363

[B42] Langendorff O Untersuchungen am uberlebenden Saugethierherzen Pflugers Arch 1895 61 291 332 10.1007/BF01812150

[B43] Skrzypiec-Spring M Grotthus B Szeląg A Schulz R Isolated heart perfusion according to Langendorff--Still viable in the new millennium J Pharmacol Toxicol Methods 2007 55 2 113 126 10.1016/j.vascn.2006.05.006 16844390

[B44] Chopra K Singh M Kaul N Ganguly NK Decrease of myocardial infarct size with desferroxamine. Possible role of oxygen free radicals in it's ameliorative effect Mol Cell Biochem 1992 113 1 71 76 1640938 10.1007/BF00230887

[B45] Marletta MA Yoon PS Iyenger R Leaf CD Wishnok JS Macrophage oxidation of L-arginine to nitrite and nitrate: Nitric oxide is an intermediate Biochem 1988 27 24 8706 8711 10.1021/bi00424a003 3242600

[B46] Szabo C Thiemermann C Vane JR Dihydropyridine modulators of calcium channel inhibit the induction of nitric oxide synthase by endotoxin in cultured J774.2 cells Biochem Biophys Res Commun 1993 196 2 825 830 10.1006/bbrc.1993.2323 7694580

[B47] Szabo C Wu CC Mitchell JA Gross SS Thiemermann C Vane JR Platelet activating factor contributes to the induction of nitric oxide synthase by bacterial lipopolysaccharide Circ Res 1993 73 6 991 999 7693362 10.1161/01.res.73.6.991

[B48] Parikh V Singh M Possible role of cardiac mast cell degranulation and preservation of nitric oxide release in isolated rat heart subjected to ischemic preconditioning Mol Cell Biochem 1999 199 1 6 10.1023/A:1006930011622 10544945

[B49] Grover GJ Dzwonczyk S Sleph PG Sergent CVR The ATP sensitive potassium channel blocker. Glibenclamide (Glyburide) does not abolish preconditioning in isolated ischaemic rat heart J Phamacol Exp Ther 1993 265 2 559 65 8496806

[B50] Fralix TA Steenbergen C London RE Murphy E Glibenclamide does not abolish the protective effect of preconditioning on stunning in the isolated perfused rat heart Cardiovasc Res 1997 27 4 630 7 10.1093/cvr/27.4.630 8324797

[B51] Kaur H Parikh V Sharma A Singh M Effect of amiloride a Na+/H+ exchange inhibitor on cardioprotective effect of ischaemic preconditioning: possible involvement of resident cardiac mast cells Pharmacol Res 1997 36 2 95 102 10.1006/phrs.1997.0174 9344636

[B52] Kersten JR Toller WG Gross ER Pagel PS Warltier DC Diabetes abolishes ischemic preconditioning: role of glucose, insulin, and osmolality Am J Physiol Heart Circ Physiol 2000 278 4 H1218 H1224 10749717 10.1152/ajpheart.2000.278.4.H1218

[B53] Wynne A Hausenloy DJ Mocanu MM Yellon DM Glimepiride reduces the threshold for ischemic preconditioning in the diabetic heart J Mol Cell Cardiol 2007 42 S171 S189

[B54] Gross ER Hsu AK Gross GJ Diabetes Abolishes Morphine-Induced Cardioprotection via Multiple Pathways Upstream of Glycogen Synthas Kinase-3β Diabetes 2007 56 1 127 136 10.2337/db06-0907 17192474

[B55] Webb A Bond R McLean P Uppal R Benjamin N Ahluwalia A Reduction of nitrite to nitric oxide during ischemia protects against myocardial ischemia-reperfusion damage PNAS 2004 101 13683 13688 10.1073/pnas.0402927101 15347817 PMC518813

[B56] Baker JE Su J Fu X Hsu A Gross GJ Tweddell JS Hogg N Nitrite confers protection against myocardial infarction: Role of xanthine oxidoreductase, NADPH oxidase and K_ATP _channels J Mol Cell Cardiol 2007 43 4 437 444 10.1016/j.yjmcc.2007.07.057 17765919 PMC2735077

[B57] Taliyan R Singh M Sharma PL Yadav HN Sidhu KS Possible involvement of α 1 adrenergic receptor and KATP channels in cardioprotective effect of remote aortic preconditioning in isolated rat heart J C D R 2010 1 3 145 151 10.4103/0975-3583.70917 PMC2982203 21187869

[B58] Baines CP Cohen MV Downey JM Signal Transduction in Ischemic Preconditioning:The Role of Kinases and Mitochondrial K_ATP _Channels: J Cardiovasc Electrophysiol 1999 10 5 741 754 10.1111/j.1540-8167.1999.tb00251.x 10355930

[B59] Murphy E Primary and secondary signalling pathways in early preconditioning that converge on the mitochondria to produce cardioprotection Circ Res 2004 94 1 7 16 10.1161/01.RES.0000108082.76667.F4 14715531

[B60] Hide EJ Thiemermann C Limitation of myocardial infarct size in the rabbit by ischaemic preconditioning is abolished by sodium 5-hydroxydecanoate Cardiovasc Res 1996 31 6 941 946 8759250

[B61] Yang M-K Lee S-H Seo H-W Yi K-W Yoo S-E Lee B-H Chung H-J Won H-S Lee C-S Kwon S-H KR-31761, a Novel K^+^ATP Channel Opener, Exerts Cardioprotective Effects by Opening Both Mitochondrial K^+^_ATP _and Sarcolemmal K^+^ATP Channels in Rat Models of Ischemia/Reperfusion-Induced Heart Injury J Pharmacol Sci 2009 109 2 222 232 10.1254/jphs.08132FP 19234365

[B62] Koneru S Penumathsa SV Thirunavukkarasu M Samuel SM Zhan L Han Z Maulik G Das DK Maulik N Redox regulation of ischemic preconditioning is mediated by the differential activation of caveolins and their association with eNOS and GLUT-4 Am J Physiol Heart Circ Physiol 2007 292 5 H2060 H2072 10.1152/ajpheart.01169.2006 17277024

[B63] Feron O Belhhassen L Kobzik L Smith TW Kelly RA Michel T Endothelial nitric oxide synthase targeting to caveolae. Specific interactions with caveolin isoforms in cardiac myocytes and endothelial cells J Biol Chem 1996 271 37 22810 22814 10.1074/jbc.271.37.22810 8798458

[B64] Sobey GC Weiler MJ Boujaoude M Woodman LO Effect of short-term phytoestrogen treatment in male rats on nitric oxide- mediated responses of carotid and cerebral arteries: comparison with 17-β estradiol J Pharmacol Exp Ther 2004 310 1 135 140 10.1124/jpet.103.063255 15054117

[B65] Woodman OL Missen MA Boujaoude M Daidzein and 17 beta-estradiol enhance nitric oxide synthase activity associated with an increase in calmodulin and a decrease in caveolin-1 J Cardiovasc Pharmacol 2004 44 2 155 163 10.1097/00005344-200408000-00003 15243295

[B66] Tsutsumi YM Kawaraguchi Y Horikawa YT Niesman IR Kidd MW Chin-Lee B Head BP Patel PM Roth DM Patel HH Role of caveolin-3 and glucose transporter-4 in isofurane induced delayed cardioprotection Anesthesiology 2010 112 5 1136 1145 10.1097/ALN.0b013e3181d3d624 20418694 PMC2860616

[B67] Osada M Takeda S Sato T Komori S Tamura K The protective effect of preconditioning on reperfusion induced arrhythmia is lost by treatment with superoxide dismutase Jpn Circ 1994 58 259 263 10.1253/jcj.58.259 8051784

[B68] Tanaka M Fujiwara H Yamasaki K Sasayama S Superoxide dis-mutase and *N*-2 mercaptopropionyl glycine attenuate infarct size limitation effect of ischemic preconditioning in the rabbit Cardiovasc Res 1994 28 980 986 10.1093/cvr/28.7.980 7954610

[B69] Roth DM Patel HH Role of caveolae in cardiac Protection Pediatr Cardiol 2011 32 3 329 333 10.1007/s00246-010-9881-8 21210089 PMC3051068

